# Targeted silencing of SOX2 by an artificial transcription factor showed antitumor effect in lung and esophageal squamous cell carcinoma

**DOI:** 10.18632/oncotarget.21523

**Published:** 2017-10-05

**Authors:** Etsuko Yokota, Tomoki Yamatsuji, Munenori Takaoka, Minoru Haisa, Nagio Takigawa, Noriko Miyake, Tomoko Ikeda, Tomoaki Mori, Serika Ohno, Takashi Sera, Takuya Fukazawa, Yoshio Naomoto

**Affiliations:** ^1^ Department of General Surgery, Kawasaki Medical School, Okayama, 700-8505, Japan; ^2^ Department of General Internal Medicine 4, Kawasaki Medical School, Okayama, 700-8505, Japan; ^3^ General Medical Center Research Unit, Kawasaki Medical School, Okayama, 700-8505, Japan; ^4^ Department of Applied Chemistry and Biotechnology, Faculty of Engineering, Okayama University, Okayama, 700-8530, Japan

**Keywords:** artificial transcription factor (ATF), SOX2, squamous cell carcinoma, molecular targeted therapy

## Abstract

*SOX2* is a transcription factor essential for early mammalian development and for the maintenance of stem cells. Recently, *SOX2* was identified as a lineage specific oncogene, recurrently amplified and activated in lung and esophageal squamous cell carcinoma (SCC). In this study, we have developed a zinc finger-based artificial transcription factor (ATF) to selectively suppress SOX2 expression in cancer cells and termed the system ATF/*SOX2*. We engineered the ATF using six zinc finger arrays designed to target a 19 bp site in the *SOX2* distal promoter and a KOX transcriptional repressor domain. A recombinant adenoviral vector Ad-ATF/*SOX2* that expresses ATF/*SOX2* suppressed SOX2 at the mRNA and protein levels in lung and esophageal SCC cells expressing SOX2. In these kinds of cells, Ad-ATF/*SOX2* decreased cell proliferation and colony formation more effectively than the recombinant adenoviral vector Ad-sh*SOX2*, which expresses *SOX2* short hairpin RNA (sh*SOX2*). Ad-ATF/*SOX2* induced the cell cycle inhibitor CDKN1A more strongly than Ad-sh*SOX2*. Importantly, the ATF did not suppress the cell viability of normal human cells. Moreover, Ad-ATF/*SOX2* effectively inhibited tumor growth in a lung SCC xenograft mouse model. These results indicate that ATF/*SOX2* would lead to the development of an effective molecular-targeted therapy for lung and esophageal SCC.

## INTRODUCTION

Lung cancer is the most frequent cause of cancer-related death world-wide [1]. Non-small cell lung cancer (NSCLC) accounts for around 85% of all lung cancers. NSCLC is comprised of two subtypes, adenocarcinoma and SCC, based on histology. Recent progress in next generation sequencing (NGS) has allowed investigators to examine the various genetic mutations within malignant tumors. The discovery of driver mutations such as epidermal growth factor receptor (EGFR) and anaplastic lymphoma kinase (ALK) has led to remarkable improvement in personalized therapy for pulmonary adenocarcinoma [2]. Further driver oncogenes have been identified in pulmonary adenocarcinoma. The identification of these mutations and amplifications can be used to predict sensitivity to clinical inhibitors of pulmonary adenocarcinoma [3]. Recently, genetic alterations in lung SCC have also been investigated broadly and genetic alterations related to lung SCC have been reported [4]. Molecular targeted therapies have taken a step forward with the discovery of these driver oncogenes for the treatment of lung SCC and several agents are expected to be effective for the treatment of the disease [5]. SCC is the predominant form of esophageal carcinoma. More than 450,000 people worldwide are suffering from esophageal cancer and the incidence is rapidly increasing. The overall 5-year survival rate for esophageal cancer is 15% to 30% and patients diagnosed at earlier stages have better outcomes than those diagnosed at later stages. In order to improve the efficacy of treatment of esophageal cancer multidisciplinary treatments have been conducted [6, 7]. In addition to the technical advance of minimally invasive surgery and endoscopic treatment, molecularly targeted agents have a key role in improving outcome. Molecular targeting agents containing small molecules and antibodies developed on the basis of molecular biology are being incorporated into multimodal therapies [8, 9], however effective molecular targeting therapies for esophageal cancer have not been established yet.

**Figure 1:**
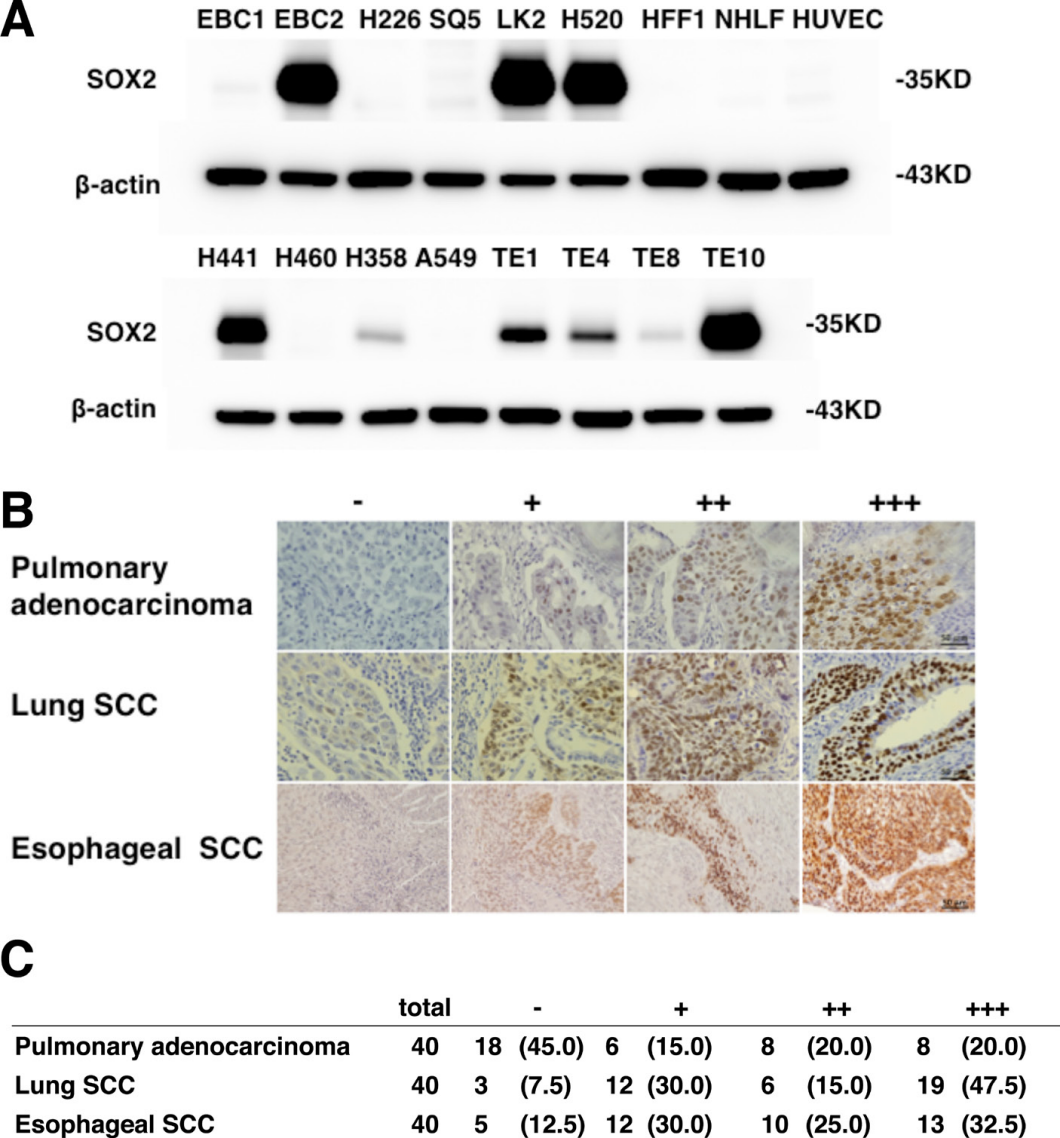
SOX2 expression in lung, esophageal SCC and pulmonary adenocarcinoma (**A**) Immunoblot analysis of SOX2 in indicated cells. The expression level of β-actin is shown as a control. (**B**) The intensity of SOX2 staining was assigned the following scores: none = -, weak = +, moderate = ++, and strong = +++ expression. Examples of representative immunohistochemistry results are shown. Bars, 50 μm. (**C**) SOX2 expression in primary pulmonary adenocarcinoma samples of 40 patients, lung SCC samples of 40 patients and esophageal SCC samples of 40 patients who underwent surgical tumor resection at the Kawasaki Hospital Attached to Kawasaki Medical School between 2007 and 2012. Percentage values are given in parentheses.

*SOX2* is a transcription factor that is fundamental for early development and for the maintenance of stem cells in multiple adult tissues and also plays an important role in squamous cell differentiation [10, 11]. Amplification of chromosome 3q26 is the most common of the genetic alterations found in lung SCC [12]. *SOX2* is a candidate oncogene present in this locus and amplification of *SOX2* has been reported in lung and esophageal SCC [13]. Bass et al. showed that inhibition of *SOX2* suppresses cell growth. There is a data that supports a role for this gene as a “lineage survival oncogene” [14]. In a previous study, we demonstrated that silencing SOX2 by siRNA induced G1 cell cycle arrest mediated by upregulation of CDKN1A expression resulting in an anti tumor effect in SOX2-expressing lung SCC cells both *in vitro* and *in vivo* [15]. As shown in Figure 1A, SOX2 expression was detected in 3 of 6 kinds of lung SCC cells, in 2 of 4 kinds of pulmonary adenocarcinoma cells and in all kinds of esophageal SCC cells, while expression of SOX2 was not detected in normal human foreskin fibroblast HFF1 and normal human lung fibroblast NHLF and human umbilical vein endothelial cells (HUVEC). In human lung SCC and esophageal SCC sections, SOX2 expression was detected in more than 87.5% of sections (Figure 1B and 1C) suggesting that molecular targeting of SOX2 might be useful for treating SCC.

**Figure 2:**
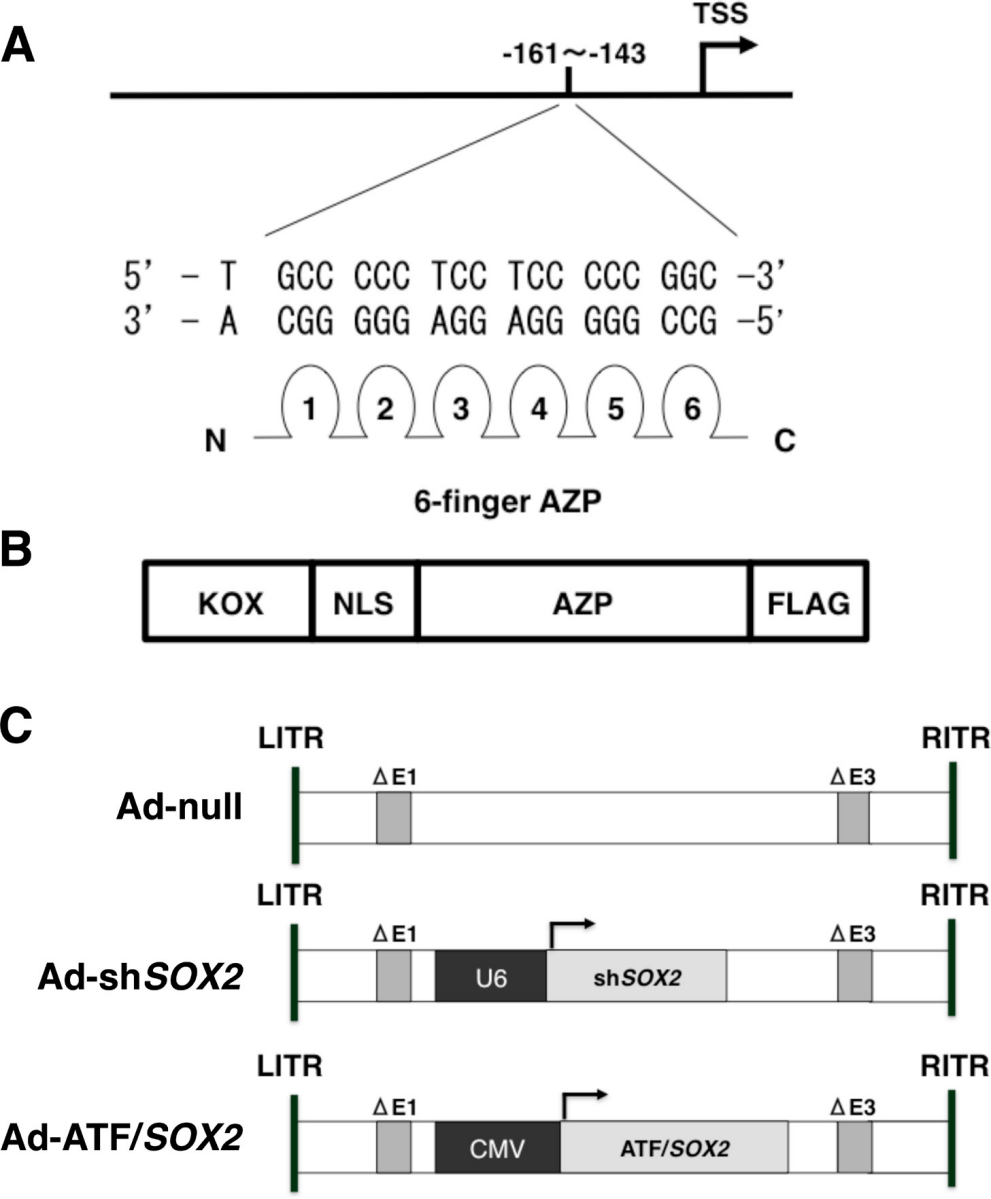
Schematic representation of ATF/*SOX2* (**A**) Design of Artificial zinc finger protein (AZP) to target a 19 bp sequence (-161: TGCCCCCTCCTCCCCCGGC:-143) in human *SOX2* distal promoter region. TSS; transcription start site. (**B**) The Artificial Transcription Factor (ATF) contains the KOX suppressor domain, a nuclear localization signal (NLS), the Artificial zinc finger protein (AZP) and a FLAG tag. We termed this ATF as ATF/*SOX2*. (**C**) Schematic representation of Ad-null, Ad-sh*SOX2* and Ad-ATF/*SOX2.* The PCR-generated expression cassette of ATF/*SOX2* from pcDNA3.1 ATF/*SOX2* or sh*SOX2* from pBAsi-mU6 sh*SOX2* (described in Materials and Methods section) were subcloned into the linearized E1 deleted adenovirus type 5 genome. E1; Adenovirus early region 1, E3; Adenovirus early region 3, LITR; Left Inverted Terminal Repeat, RITR; Right Inverted Terminal Repeat.

As we reviewed previously, zinc finger -based artificial transcription factors (ATFs) [16] can be designed to regulate the expression of target genes and can provide powerful biotechnological tools for the investigation and treatment of disease [17, 18]. We demonstrated that a simple mode of DNA recognition by zinc finger domains makes it possible to design ATFs with novel sequence specificities [19]. The designed ATFs can perform DNA-binding activity and conduct natural transcription and they have been used to either activate or repress miscellaneous endogenous target genes [20–22]. We have also demonstrated that designed regulatory proteins (DRPs), in which artificial transcription factors (ATFs) are fused to cell-penetrating peptides (CPPs) effectively activate or repress target genes [23]. In a previous study, Stolzenburg et al. developed zinc finger-based artificial transcription factors (ATFs) to target SOX2 and showed their possible therapeutic use against breast cancer [24]. In order to target SOX2 as a candidate oncogene of lung SCC and esophageal SCC, we have developed a zinc finger-based artificial transcription factor (ATF) to selectively suppress SOX2 expression in cancer cells. We termed this system ATF/*SOX2* (Figure 2A and 2B). Ad-ATF/*SOX2* (Figure 2C) up-regulated *CDKN1A* mRNA and protein expression more significantly than did Ad-sh*SOX2* in SOX2-expressing lung and esophageal SCC cells *in vitro*. Furthermore, Ad-ATF/*SOX2* induced an antitumor effect in SOX2-expressing SCC more effectively than did Ad-sh*SOX2* both *in vitro* and *in vivo*. These results indicate that the transcriptional SOX2 inhibition achieved by ATF/*SOX2* activated CDKN1A and showed a greater antitumor effect more strongly than post-transcriptional SOX2 inhibition by shRNA. Here we report a novel SOX2-targeting therapy using an ATF for the treatment of lung and esophageal SCC.

## RESULTS

### ATF/*SOX2* suppressed *SOX2* transcriptional activity and protein expression in lung and esophageal SCC cells

**Figure 3:**
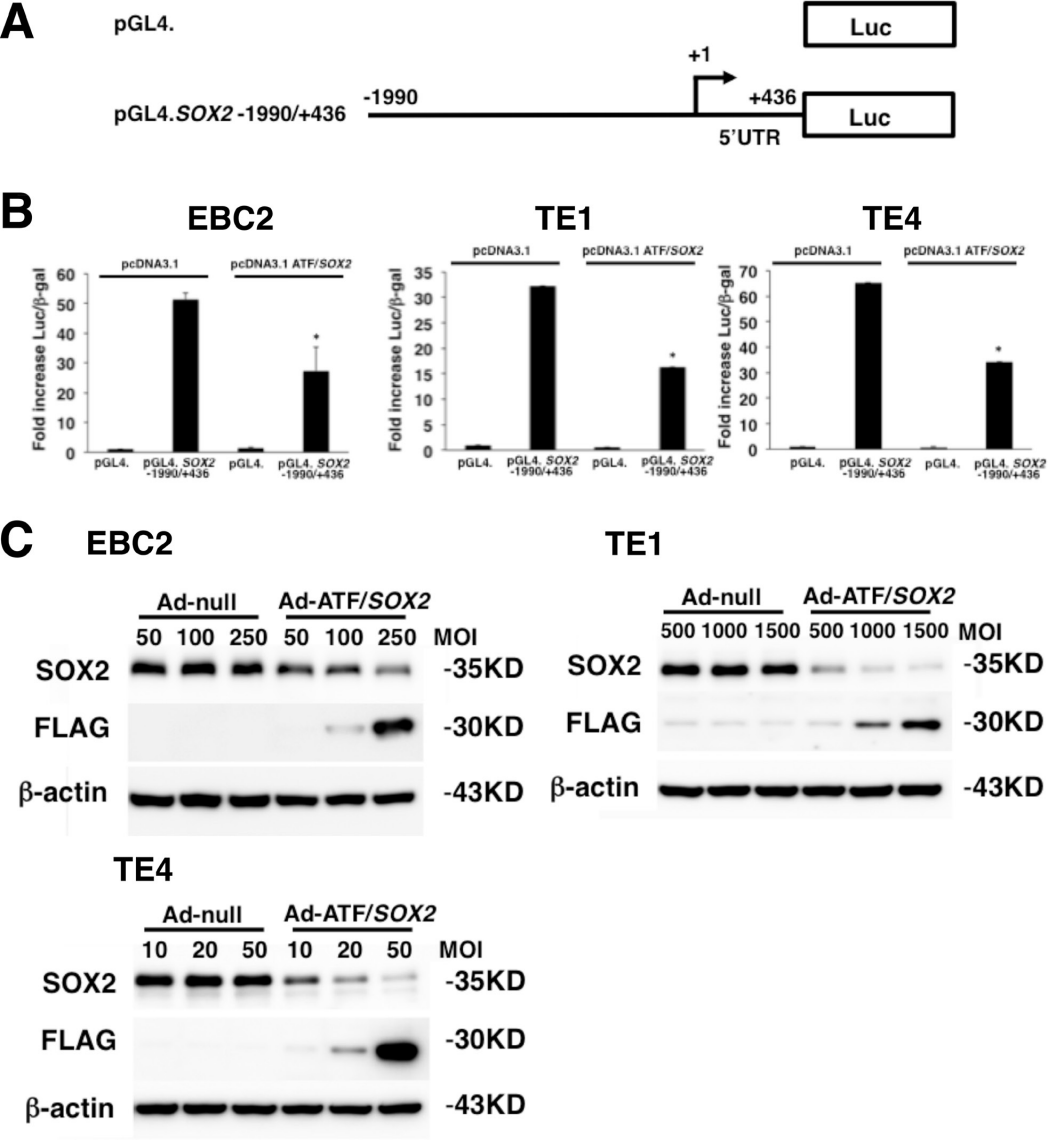
Repression of *SOX2* transcriptional activity and protein expression by ATF/*SOX2* in lung and esophageal SCC cells (**A**) Schematic representation of *SOX2* distal and proximal promoter reporter constructs. TSS; transcription start site. Luc; Luciferase. (**B**) Transient transfection reporter assays in lung SCC cells and esophageal squamous carcinoma cells with the indicated luciferase reporter constructs (2 μg, pGL4), effector constructs (2 μg, pcDNA3.1) and pCMV. β-gal (1 μg). Results are presented as fold induction of relative light units normalized to β-galactosidase activity relative to that observed for control constructs. Statistical analysis was performed using Student’s *t* test (two-tailed, unpaired). Statistical significance was defined as ^*^p < 0.01 vs pcDNA3.1 transfected group. (**C**) Ad-ATF/*SOX2* dose dependently increased ATF/*SOX2* fused to the FLAG epitope and suppressed SOX2 expression in EBC2 cells, TE1 and TE4 cells 48 hours after infection. SOX2 expression was not changed after Ad-null infection in these kinds of cells.

In order to suppress SOX2 expression in lung SCC, we have generated a zinc finger-based artificial repressor for SOX2 termed ATF/*SOX2*. We engineered ATF/*SOX2* using six zinc finger arrays designed to bind a 19 bp site in the *SOX2* distal and proximal promoter region [24]. The six zinc finger domains are linked to the nuclear localization signal (NLS) and KOX (zinc finger 10) repressor domain (Figure 2A, 2B). To test the ability of ATF/*SOX2* to suppress SOX2 expression we measured *SOX2* transcriptional activity in lung SCC cells in the presence or absence of ATF/*SOX2* cloned into the mammalian expression vector pcDNA3.1. As shown in Figure 3A and 3B, in the control, which lacked ATF/*SOX2*, the *SOX2* distal and proximal promoter and 5’UTR region –1990/+436 exhibited significant transcriptional activity in EBC2 SCC cells (52 -fold) 24 hours after transfection. On the other hand, in the presence of pcDNA3.1 ATF/*SOX2*, transcriptional activity of the *SOX2* was significantly decreased in EBC2 cells (1.9-fold). In esophageal SCC cells, the region –1990/+436 exhibited significant transcriptional activity in TE1 cells (32 -fold) and in TE4 cells (65 -fold). ATF/*SOX2* suppressed significantly the transcriptional activity in TE1 cells (1.9 -fold) and in TE4 cells (1.9 -fold). These results indicate that ATF/*SOX2* significantly suppresses SOX2 transcriptional activity in lung and esophageal SCC cells.

Next, we evaluated whether *SOX2* expression could be suppressed by adenoviral mediated ATF/*SOX2* (Ad-ATF/*SOX2,* Figure 2C) induction in lung and esophageal SCC cells. Immunoblot analysis was performed using anti-FLAG antibody in order to detect ATF/*SOX2* expression. As shown in Figure 3C, Ad-ATF/*SOX2* dose dependently increased FLAG tagged ATF/*SOX2* expression in EBC2 lung SCC cells, TE1 and TE4 esophageal SCC cells 48 hours after infection. Ad-ATF/*SOX2* suppressed SOX2 expression in these kinds of cells in a dose dependent manner. FLAG-tagged ATF/*SOX2* was not induced and SOX2 expression was not changed after Ad-null (Figure 3C) infection in the cells.

### ATF/*SOX2* induced CDKN1A expression more strongly than sh*SOX2* in SOX2-expressing lung and esophageal SCC cells

**Figure 4:**
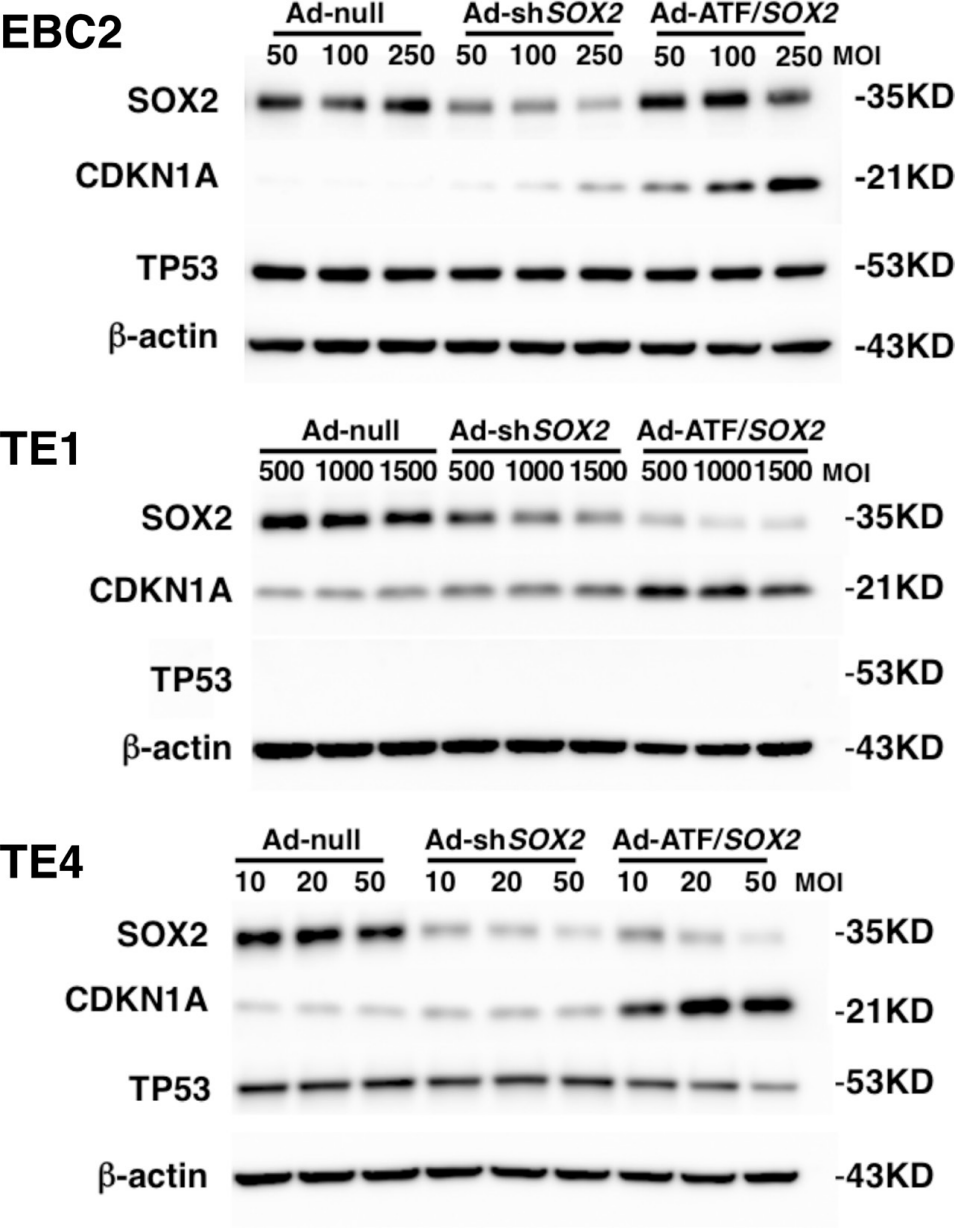
Ad-ATF/*SOX2* up-regulated CDKN1A in lung and esophageal SCC cells more robustly than Ad-sh*SOX2* Immunoblot analysis shows Ad-ATF*/SOX2* increased CDKN1A expression more than Ad-sh*SOX2* 48 hours after adenoviral infections in EBC2 lung SCC cells, TE1 and TE4 esophageal SCC cells.

We previously reported that *CDKN1A* is a *SOX2* downstream gene and that silencing of SOX2 increases the expression of CDKN1A which induces cell cycle arrest in lung SCC cells [15]. In order to analyze the antitumor effect induced by ATF/*SOX2* we transfected SCC cells with Ad-ATF/*SOX2*. We also used sh*SOX2* in an adenoviral vector (Ad-sh*SOX2*) to compare the efficacy of ATF/*SOX2* and sh*SOX2* inhibition of *SOX2* expression. We then determined *SOX2* and *CDKN1A* expression in lung and esophageal SCC cells after Ad-sh*SOX2* or Ad-ATF/*SOX2* infection. The optimal multiplicity of infection (MOI) was determined by infecting each cell line with Ad-CMV/*GFP* and choosing the MOI in which over 80% of the cells were infected [25]. As shown in Figure 4, both Ad-sh*SOX2* and Ad-ATF/*SOX2* effectively suppressed SOX2 expression in EBC2 lung SCC cells, TE1 and TE4 esophageal SCC cells 48 hours after adenoviral infections. Importantly, in EBC2 cells and TE4 cells, SOX2 protein expression was more robustly suppressed by Ad-sh*SOX2* than by Ad-ATF/*SOX2,* however, *CDKN1A* expression was greater in all kinds of cells in the Ad-ATF*/SOX2* treated cells than in Ad-sh*SOX2* treated cells. qPCR analysis also showed that Ad-ATF/*SOX2* induced *CDKN1A* mRNA more significantly than Ad-sh*SOX2* in these kinds of cells (Supplementary Figure 1). Moreover, as shown in Supplementary Figure 2, G1 cell cycle arrest was induced in EBC2 cells and TE4 cells 36 hours after Ad-ATF/*SOX2* infection, whereas the G0/G1 cell population was very weakly increased after Ad-sh*SOX2* infection. *CDKN1A* is known as one of the downstream genes of *TP53*. In this study, EBC2 lung SCC cells, TE1 and TE4 esophageal SCC cells harbor mutant *TP53.* TP53 expression was not altered after Ad-ATF*/SOX2* infection in lung and esophageal squamous SCC cells, suggesting that ATF/*SOX2* induces *CDKN1A* in *TP53* independent manner (Figure 4) [15].

### Inhibition of SOX2 by Ad-ATF/*SOX2* suppressed cell viability and colony formation of SOX2-expressing lung and esophageal SCC cells

**Figure 5:**
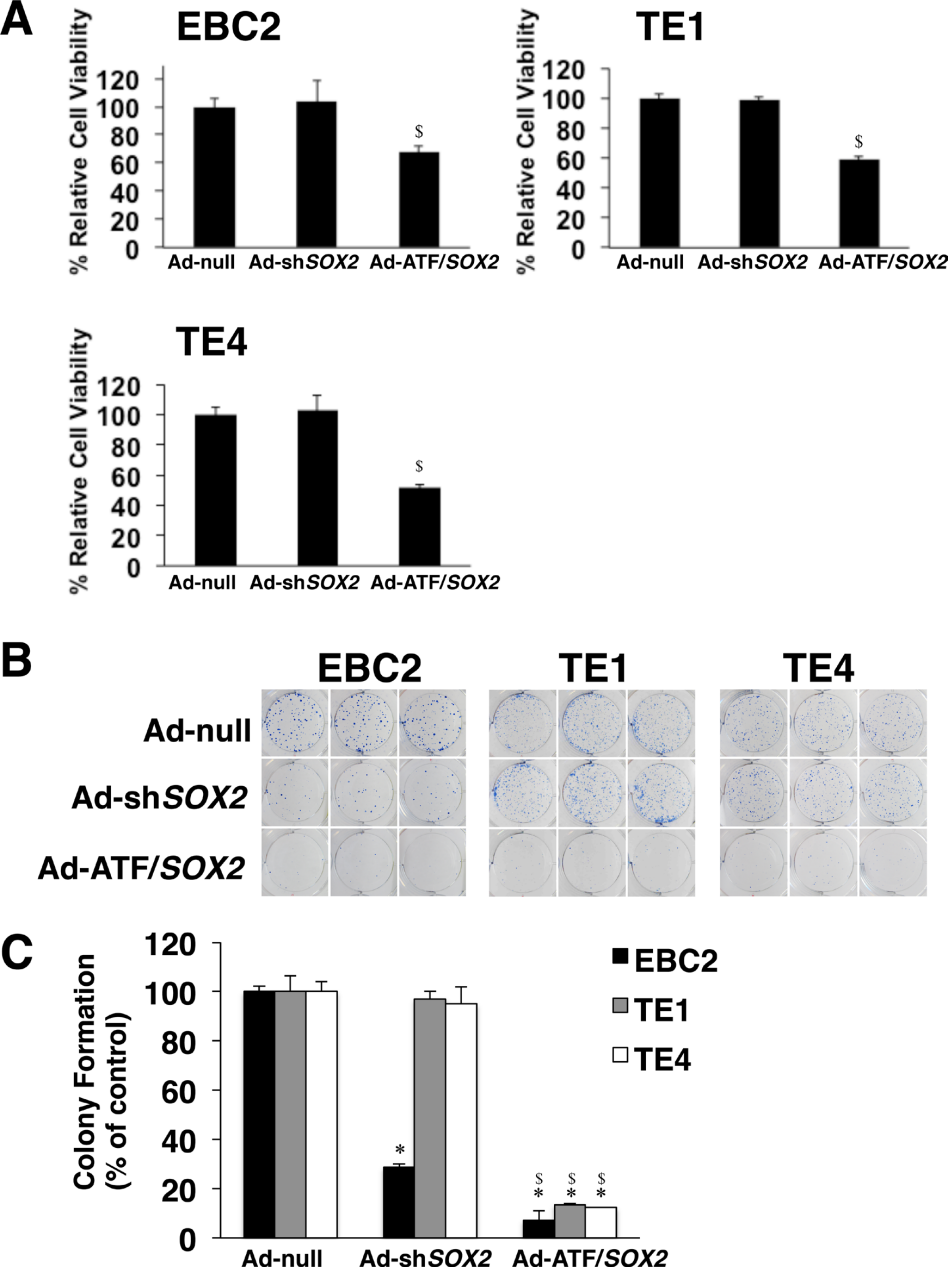
Ad-ATF/*SOX2* significantly decreases cell viability and colony formation of SOX2-expressing lung and esophageal SCC cells (**A**) Ad-ATF/*SOX2* more significantly inhibited cell growth of SOX2-expressing EBC2 lung SCC cells and TE1, TE4 esophageal SCC cells than Ad-sh*SOX2*. Cell viability was assessed 48 hours after adenoviral infection with a TC20 automated cell counter. Results represent the mean ± SD (*n* = 3). Statistical analysis was performed using Student’s *t* test (two-tailed, unpaired). Statistical significance was defined as ^$^*p* < 0.01 vs Ad-sh*SOX2* treated group. (**B**) Colony formation of EBC2 lung SCC cells, TE1 and TE4 esophageal SCC cells treated with Ad-null, Ad-sh*SOX2* or Ad-ATF/*SOX2*. 7 to 14 days after treatment, cells were fixed and stained with Diff-Quik. Representative pictures of experiments performed in triplicate are shown. (**C**) Mean colony number was derived from quantitation of triplicate dishes for each treatment and was arbitrarily set to 100%. Data are shown relative to the control group. Results represent the mean ± SD (*n* = 3). Statistical analysis was performed using Student’s *t* test (two-tailed, unpaired). Statistical significance was defined as ^*^p < 0.01 vs Ad-null treated group at the same MOI; ^$^*p* < 0.01 vs Ad-sh*SOX2* treated group at the same MOI.

In order to elucidate the antitumor effect of the ATF for *SOX2*, we analyzed cell viability and colony formation of lung SCC cells after Ad-ATF/*SOX2* infection. 48 hours after infection Ad-ATF/*SOX2* significantly suppressed the cell proliferation of EBC2 lung SCC cells, TE1 and TE4 esophageal SCC cells compared to Ad-null and Ad-sh*SOX2* (Figure 5A). Furthermore, as shown in Figure 5B and 5C, Ad-ATF/*SOX2* significantly decreased colony formation more than Ad-null and Ad-sh*SOX2* in EBC2, TE1 and TE4 cells. These results indicate that Ad-ATF/*SOX2* shows an antitumor effect more strongly than Ad-sh*SOX2* in all the SOX2 positive lung and esophageal SCC cells indicated. In this experiment, Ad-sh*SOX2* inhibited colony formation of EBC2 cells but not of TE1 cells and TE4 cells. This is concordant with the results shown in Figure 4. There was little difference in CDKN1A expression after Ad-null and Ad-sh*SOX2* infection in TE1 cells and TE4 cells. On the other hand, CDKN1A expression was clearly different after Ad-null and Ad-sh*SOX2* infection in EBC2 cells. It is possible that Ad-sh*SOX2* could not significantly inhibit cell viability in EBC2 lung SCC cells just 48 hours after infection but that it could show anti tumor effect in colony formation of EBC cells during a longer time incubation after treatment but not in TE1 and TE4 cells.

### Down- regulation of SOX2 suppresses lung SCC growth in a xenograft mouse model

**Figure 6:**
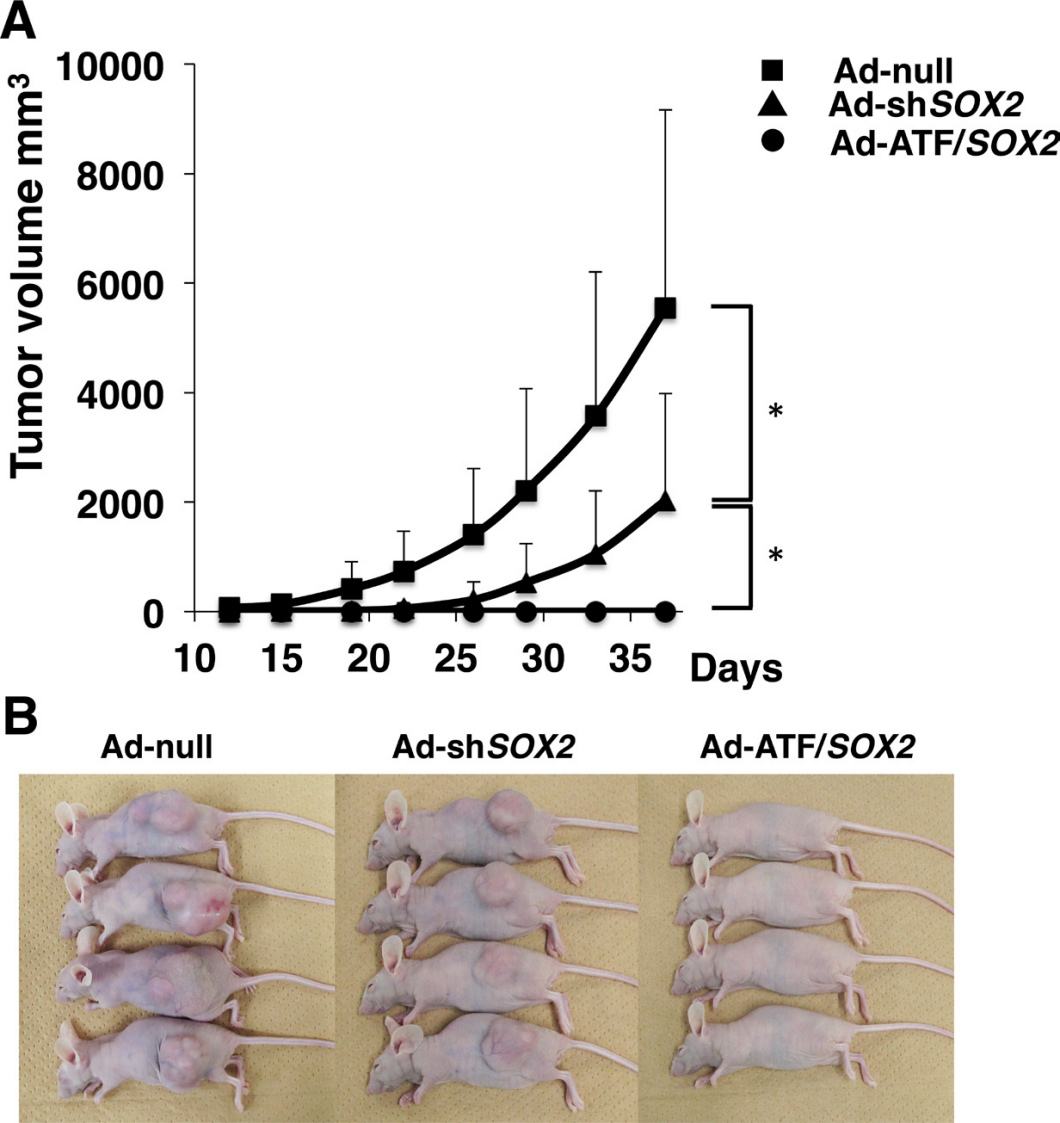
Effect of EBC2 xenograft tumor volumes as a function of time and treatment with adenoviral vectors (**A**) Volume of the tumors derived from EBC2 lung SCC cells treated with Ad-null, Ad-sh*SOX2* or Ad-ATF/*SOX2* at a MOI of 250 is shown. The volume was monitored over time (days) after inoculation of tumor cells. Fifteen mice were studied in each group. Tumor growth is expressed as mean tumor volume; bars represent SD. Statistical analysis was performed using Student’s *t* test (two-tailed, unpaired). Statistical significance was defined as ^*^p < 0.01. (**B**) Macroscopic appearance of EBC2 lung SCC tumors in xenograft mice at 39 days after inoculation.

In addition to the cell-based experiments, we used an EBC2 lung SCC xenograft nude mice tumor model to determine whether ATF/*SOX2* suppresses tumor growth *in vivo*. The tumor volume in the mice group treated with Ad-sh*SOX2* was approximately 37% of those in the mice group treated with Ad-null (*p = 0.0026*). Importantly, Ad-ATF/*SOX2* completely inhibited the tumor growth (*p = 0.00039 vs* Ad-sh*SOX2* treated group, Figure 6A and 6B). These results indicate that Ad-ATF/*SOX2* significantly induced an antitumor effect against SCC more than Ad-sh*SOX2 in vivo*.

## DISCUSSION

In this study, we have shown both *in vitro* and *in vivo* that the targeted down-regulation of SOX2 using ATF based technologies can be used as an effective tool for the treatment of SCC in lung and esophageal cancers that express SOX2. We showed that ATF/*SOX2* up-regulated *CDKN1A*, one of the target genes of *SOX2*, and induced cell G1 cycle arrest more effectively than sh*SOX2* in SOX2-expressing EBC2 lung cells and TE4 esophageal SCC cells (Figure 4, Supplementary Figure 1, Supplementary Figure 2). In addition, the ATF more significantly suppressed cell viability and colony formation of SOX2-expressing lung and esophageal SCC cells compared to sh*SOX2,* whereas little CDKN1A expression was induced after Ad-ATF/*SOX2* infection in SOX2 negative lung SCC cells and normal human cells HUVEC and NHLF (Supplementary Figure 3A). Cell viability was not significantly suppressed in these kinds of normal cells after Ad-ATF/*SOX2* treatment, compared to control vector (Supplementary Figure 3B). These results indicate that Ad-ATF/*SOX2* could induce antitumor effect in SCC cells expressing SOX2 but not in normal cells. Each zinc finger domain specifically recognizes 3 or 4 bp of DNA and ATF/*SOX2* recognizes a 19 bp sequence in close proximity to the transcriptional start of the human *SOX2* distal and proximal promoter region, providing a high degree of specificity. It is theoretically necessary to recognize at least 16 bp of DNA for specific recognition of one genomic region in human cells based on a genome size of 3 × 10^9^ bp. The Basic Local Alignment Search Tool (BLAST) on the human genome revealed that the 19 bp target sequence in the human *SOX2* distal promoter region is unique. Ad-ATF/*SOX2* induced CDKN1A expression in all three kinds of SOX2-expressing lung and esophageal SCC cells but not in all five kinds of SOX2 negative cells (Figure 4, Supplementary Figure 3A). Moreover, as shown in Supplementary Figure 4, Ad-ATF/*SOX2* did not change mRNA expression of ABL1, RTN4 and KMT2B that contained a highly similar sequence to the 19 bp target sequence of ATF/*SOX2* in EBC2 cells. In addition to this, the other kinds of zinc finger based ATFs previously reported [26] alter neither SOX2 nor CDKN1A expression in EBC2 cells (Supplementary Figure 5). These results indicate that induction of CDKN1A, the downstream targets of SOX2, after Ad-ATF/*SOX2* infection was not the result of off-target action of ATF/*SOX2* (was on-target effect of ATF/*SOX2*). In this study, SOX2 expression was more robustly suppressed by Ad-sh*SOX2* than by Ad-ATF/*SOX2* in EBC2 cells and TE4 cells. However, Ad-ATF/*SOX2* induced CDKN1A and the other *SOX2* related genes [15] more than Ad-sh*SOX2* in these kinds of cells (Figure 4, Supplementary Figure 6). We believe that this discrepancy is not the result of off-target action of ATF/*SOX2* but could possibly be due to kinetics-dependent differences of ATF and shRNA. There could also be another possibility in that the distal or proximal promoter region of *SOX2* could suppress the expression of unidentified noncoding RNA, which shRNA for *SOX2* does not target, leading to induction of *CDKN1A*. Recently, a noncoding RNA, *SOX2* overlapping transcript (*SOX2OT*), has been identified as having a role in lung and esophageal cancer [27]. *SOX2OT* is a long noncoding RNA (lncRNA) mapped to human chromosome 3q26.3-q27 where the SOX2 gene is embedded within its third intron [28]. Shahryari et al. reported that suppression of a novel splice variant of *SOX2OT* (*SOX2OT-S1*) increased the number of cells in G1 phase in human embryonal carcinoma NTERA2 cells [29]. Furthermore, the KOX transcriptional repressor domain used in the current study works as an effective repressor of distal and proximal gene regulatory elements including enhancers [30, 31], therefore ATF/*SOX2* might inhibit the expression of not only *SOX2* but also an unidentified distal noncoding RNA. Further investigation of the loci around the SOX2 gene is needed in order to elucidate this discrepancy.

In our previous investigation, we identified 15 SOX2 related genes (4 negatively-correlated and 11 positively-correlated with *SOX2*) in lung SCC cells using RNA-seq data from 178 lung SCC specimens [32] and another RNA-seq dataset from 105 non-small cell lung cancer cell lines [4]. As shown in Supplementary Figure 6, the ATF suppressed *MSH6* mRNA and protein expression in EBC2 cells, TE4 cells and TE10 cells suggesting that these genes might be downstream genes of SOX2 in lung and esophageal SCC. On the other hand, the PI3 Kinase inhibitor wortmannin suppressed SOX2 expression in EBC2 lung SCC cells and in TE1, TE10 esophageal SCC cells whereas ATF/*SOX2* did not alter phosphorylated AKT (pAKT) expression, suggesting that pAKT might be an upstream regulator of SOX2 in these kinds of cells (Supplementary Figure 7) [33, 34]. Importantly, AKT and β-actin expression were not altered after wortmannin treatment. However, further investigation is needed to clarify whether pAKT directly regulates SOX2 or not. In this experiment, we used TE10 esophageal SCC cells because TE4 esophageal SCC cells have little PI3 Kinase expression (data not shown). Thus a combination treatment of the ATF targeting *SOX2* and PI3 Kinase inhibitor might prove to be an effective antitumor treatment for esophageal and lung SCC (Supplementary Figure 8).

The technology of gene activation / repression with zinc finger (ZF)-based artificial transcription factors (ATFs), TAL Effector or CRISPR a/i are evolving rapidly [35–37]. RNAi is known as the most universally used gene repression technology and is categorized as either small interfering RNAs (siRNA) or short hairpin RNAs (shRNA) [38]. To achieve repression, RNAi requires only a single small component, while other systems need 2 or 3 components (e.g.: dCas9, sgRNA and repression module) that are difficult to deliver [36, 37]. On the other hand, there is concern that RNAi may have many off-target effects and reproducibility issues [39]. Compared to RNAi, off-target effects with the use of ATFs, TALE and CRISPR intervention is considered to be minimal [40]. Furthermore, the molecular weight of ATFs is much smaller than TAL effector and CRISPR a/i thus ATFs may have an advantage in delivery to target cells over the other systems.

Delivery of therapeutic viral vectors into the target organ and tumor is required for the treatment of lung cancer or esophageal cancer [41–43]. One of the drawbacks of this approach is the lack of means to effectively deliver the therapeutic virus to target different kinds of cells that reside within an intricate lung and esophageal structure. However, we have previously demonstrated that the ATFs fuse to cell-penetrating peptides (CPPs) and effectively activate or repress target genes *in vitro* [23]. Importantly, this approach does not depend on cell types and could prevent the risk of insertional mutagenesis and cause relatively fewer off-target effects than ATF gene delivery systems that rely on expression from DNA and mRNA [44]. Thus, CPPs fused to ATF might be feasible treatments to target lung and esophageal SCC in the near future.

Although several technical challenges and uncertainties remain, further advances in understanding and improvements in ATF technology will open the next era of cancer therapy. In this study we have used ATF technology to successfully suppress SCC *in vitro* and *in vivo* in SOX2-expressing SCC lung and esophageal cancer cells as a first step in the search for an effective treatment for SCC lung and esophageal cancers.

## MATERIALS AND METHODS

### Cell lines and culture conditions

The human lung SCC cells H520, H226, the human pulmonary adenocarcinoma cells H358, H441, H460 and A549, the human esophageal SCC cells TE1, TE4, TE8, TE10 and human foreskin fibroblast HFF1 obtained from the American Type Culture Collection (Manassas, VA) and the normal human lung fibroblast cells NHLF obtained from Lonza (Portsmouth, NH) were grown in RPMI 1640 (H226, H358, H460, H520, TE1, TE4, TE8, TE10) or high glucose Dulbecco’s modified Eagle medium (H441, A549, HFF1, NHLF) supplemented with 10% heat-inactivated fetal bovine serum. Human umbilical vein endothelial cells (HUVEC) were purchased and grown in Endothelial Cells Growth Medium (Medium 200) supplemented with Low Serum Growth Supplement kit (Thermo Fisher Scientific, Rockford, IL). The lung squamous cell carcinoma cells EBC1, EBC2, SQ5, LK2 were kindly provided by Dr. Kiura Katsuyuki (Department of Respiratory Medicine, Okayama University Graduate School of Medicine and Dentistry, Okayama, Japan) and grown in RPMI 1640 supplemented with 10% heat-inactivated fetal bovine serum. All cell lines were cultured in 5% CO_2_ at 37°C.

### Immunohistochemistry

Sections were sequentially deparaffinized through a series of xylene, graded ethanol, and water immersion steps. After being boiled in target retrieval solution (Dako, Carpinteria, CA, USA) for 15 minutes, sections were incubated with 3% hydrogen peroxide for 5 minutes to block endogenous peroxidase activity. A primary antibody specific for human SOX2 was obtained from Cell Signaling Technology (Beverly, MA). Specimens were incubated overnight at 4°C with a 1:100 dilution of antibody followed by three washes with TBS. The slides were treated with streptavidin-biotin complex (Envision System labeled polymer, horseradish peroxidase [HRP], Dako, Carpinteria, CA) for 60 minutes at room temperature. Immunoreactions were visualized using a 3,3′-diaminobenzidine (DAB) substrate-chromogen solution (Dako Cytomation Liquid DAB Substrate Chromogen System, Dako) and counterstained with hematoxylin. Sections were immersed in an ethanol and xylene bath and mounted for examination. For immunohistochemistry analysis, 40 lung SCC tissue samples, 40 pulmonary adenocarcinoma tissues samples and 40 esophageal SCC samples in tissue sections were obtained from patients diagnosed with lung SCC and pulmonary adenocarcinoma and who underwent surgical resection at Kawasaki Hospital, Okayama, Japan. The experimental protocol was approved by the Ethics Review Committee of Kawasaki Medical School (Ethics Committee reference number: 1310).

### Design and construction of ATF/*SOX2*

We designed and constructed an artificial zinc finger protein (AZP) targeting –161 to –143 in the human SOX2 gene, where +1 is the transcription start site, by using our recognition code table as described [19]. The DNA encoding the AZP was cloned into pcDNA3.1+ (Thermo Fisher Scientific) containing a Krüppel-associated box domain of KOX1; a nuclear localization signal from the simian virus 40 large T antigen; and a FLAG epitope tag to construct the ATF-expression vector pcDNA3.1 ATF/*SOX2*.

### Adenoviral vectors

A plasmid vector expressing sh*SOX2*: pBAsi-mU6 sh*SOX2* was constructed by ligating a shRNA sequence for *SOX2* (GCTCTTGGCTCCATGGGTT) into pBAsi-mU6 Pur (Takara Bio Inc. Otsu. Japan). The recombinant adenoviral vector Ad-sh*SOX2* was generated by subcloning the expression cassette of mU6 sh*SOX2* from pBAsi-mU6 sh*SOX2* using PCR primers (5′- GTAACTATAACGG TCATGTGGTATGGCTGATTATGATCGAATCG and 5′- ATTACCTCTTTCTCCTAAAACGACGGCCAGTGCCAAGC). Ad-ATF/*SOX2* was generated by subcloning the expression cassette of ATF/*SOX2* from pcDNA3.1 ATF/*SOX2* using PCR primers (5′- GTAACTATAACGGTCGCGATGTACGGGCCAGATATAC and 5′- ATTACCTCTTTCTCCCTGGTTCTTTCCGCCTCAGA). These PCR-generated fragments were directly subcloned into the linearized pAdenoX vector using Adeno-X Adenoviral System 3 Universal according to manufacture’s protocol (Takara Bio Inc.). The viral titer for each vector was determined by the Adeno-X™ Rapid Titer Kit (Takara Bio Inc.) and the optimal multiplicity of infection (MOI) was determined by infecting each cell line with Ad-CMV/*GFP* and assessing the expression of GFP [25].

### Luciferase reporter construct and transient transfection reporter assay

The *SOX2* proximal and distal promoter region of was obtained from human genomic DNA (Invitrogen, Life Technologies, Carlsbad, CA). The position of the transcription initiation site (+1) was determined by the Ensembl Human Genome browser. The luciferase reporter construct: pGL4. *SOX2* –1990/+436 was generated by subcloning the *SOX2* promoter region –1990/+436 amplified from the genomic DNA using PCR primers (5′-ttt^Nhe1^GCTAGC^-1990^acaagccataacttgagagaaaaaggagaaccttc and 5′-aaa^HindIII^aagctt ^+436^gcgggcgctgtgcgcgggcccggcccgccggcggc), digested with NheI and HindIII and subcloned into pGL4.23 luciferase reporter construct (Promega. Madison. WI.). All of the transfections were carried out in 6-well plates. Cells were seeded 1 day before transfection at a density of 2 × 10^5^ per well. Transfections were carried out with Lipofectamine 3000 (Thermo Fisher Scientific) in accordance with the manufacturer’s protocol as indicated. Transfected cells were harvested at 24 hours. Results of one representative experiment are presented as fold induction of relative light units normalized to β-galactosidase activity relative to that observed for the control vectors. Each experiment was repeated at least three times. Error bars indicate the SD from the average of the triplicate samples in one experiment.

### Cell viability assay

EBC2 lung SCC cells were plated at a density of 1 × 10^5^ cells per well, TE1 esophageal SCC cells were plated at a density of 2 × 10^5^ cells and TE4 esophageal SCC cells were plated at a density of 4 × 10^5^ cells in a 6-well plates and cultured overnight at 37°C. The following day Ad-null, Ad-sh*SOX2* or Ad-ATF/*SOX2* was infected at a MOI of 250 for EBC2 cells, at a MOI of 1500 for TE1 and at a MOI of 50 for TE4. Cells were harvested 48 hours after adenoviral infection and viable cells were assessed by a TC20 automated cell counter (Bio-Rad, Hercules, CA). [45].

### Co-transfection and puromycin selection

Cells were transfected using Lipofectamine 3000 (Thermo Fisher Scientific) according to manufacturer’s protocol. Cells were seeded into 6-well plates at a density of 2.5 × 10^5^ per well 1 day before transfection. To enrich for transfected cells, plasmid with puromycin resistant cassette pPUR (0.5 μg, Takara Bio, Inc.) was co-transfected with pcDNA3.1, pGFPZFN1.4-B2H, pGFPZFN2-B2H, pST1374, pPIGAZFN-L1 and pPIGAZFN-R2 (2 μg, obtained from Addgene, Cambridge, MA) [26]. After 24 hours, cells were selected in 2 μg/ml puromycin for 48 hours, then cells were harvested and the protein was isolated according to the manufacturer’s instructions.

### Colony formation assay

EBC2 cells were first plated at a density of 1 × 10^5^ cells, TE1 cells were plated at a density of 2 × 10^5^ cells and TE4 cells were plated at a density of 4 × 10^5^ cells per well in 6-well plates 24 hours before virus infection. The following day Ad-null, Ad-sh*SOX2* or Ad-ATF/*SOX2* were infected at a MOI of 250 for EBC2 cells at a MOI of 1500 for TE1and at a MOI of 50 for TE4 cells for 24 hours. EBC2 cells were released from the dish by incubation with trypsin/EDTA, counted, plated in triplicate at a density of 1 × 10^3^ cells in 6-well plates for 7 days. TE1 cells were plated in triplicate at a density of 2 × 10^3^ cells in 6-well plates for 8 days. TE4 cells were plated in triplicate at a density of 3 × 10^3^ cells in 6-well plates for 14 days. The cells were fixed and stained with Diff-Quik (Sysmex, Kobe, Japan) [46]. Colonies (a group of aggregated cells numbering at least 50) were then counted [47]. The mean number of the control group was arbitrarily set to 100%, and all other numbers were normalized and percentage-specific cytotoxicity compared to colony formation in the control group was calculated.

### Immunoblot analysis

Cells were lysed in ice-cold M-PER lysis buffer purchased from Thermo Fisher Scientific. Cell lysates were clarified by centrifugation (20 min at 15,000 rpm at 4°C) and protein concentration determined using the BCA Protein Assay (Thermo Fisher Scientific). Equal amounts of protein were separated on an SDS-PAGE gel. The gel was electrophoretically transferred to a Hybond PVDF transfer membrane (Millipore, Bedford, Massachusetts) and incubated with primary and secondary antibodies according to the Supersignal^®^ West Pico chemiluminescence protocol (Pierce, Rockford, IL). Antibody specific for FLAG (DYKDDDDK epitope) tag was obtained from Takara Bio, Inc. (Shiga, Japan) and antibody specific for SOX2, CDKN1A, VIM, AKT and phosphorylated AKT (Ser473) were purchased from Cell Signaling Technology (Beverly, MA). Antibody specific for TP53 and β-actin were obtained from Santa Cruz Biotechnology (Santa Cruz, CA) and antibody specific for MSH6 and BMP2 were purchased from Proteintech Japan (Tokyo, Japan). Secondary horseradish peroxidase-conjugated antibodies were obtained from Jackson Immunoresearch Laboratories (West Grove, PA). Each experiment was repeated at least three times and the representative data is displayed.

### Real time PCR

Total RNA from the cultured cells was obtained by using TRIzol Reagent (Thermo Fisher Scientific). 2 μg of total RNA was used for reverse transcription. Reverse transcription was performed at 37°C for 15 min using PrimeScript RT reagent Kit (Takara Bio, Inc.). The specific probe for *CDKN1A* (Hs00355782_m1), *BMP2* (Hs00154192_m1), *SNAI1* (Hs00195591_m1), *VIM* (Hs00958111_m1), *ABL1* (Hs01104728_m1), *RTN4* (Hs00199671_m1), *KMT2B* (Hs00207065_m1), *MSH6* (Hs00943000_m1) and *Glyceraldehyde-3-phosphate dehydrogenase* (*GAPDH*) (Hs03929097_g1) were derived from the commercially available TaqMan Gene Expression Assays (Applied Biosystems, Life Technologies, CA). The real-time PCR reactions were carried out in a 48-well microtiter plate using the TaqMan Gene Expression Master Mix (TaqMan One-Step RT-PCR kit) (Applied Biosystems). All samples were analyzed in triplicate in three independent experiments. The fluorescence of the PCR products was detected by the same apparatus. The number of cycles for the amplification plot to reach the threshold limit (Ct value) was used for quantification. GAPDH was used as endogenous control.

### Flow cytometric analysis for cell cycle

For cell cycle analysis, EBC2 cells were plated at a density of 1 × 10^5^ cells, TE4 cells were plated at a density of 4 × 10^5^ cells per well in 6-well plates and cultured overnight at 37°C. The following day Ad-null, Ad-sh*SOX2* or Ad-ATF/*SOX2* was infected at a MOI of 250 for EBC2 cells and at a MOI of 50 for TE4. 36 hours after infection, cells were harvested and washed once with PBS. Cells were resuspended in PBS containing 0.1 % Triton X-100 and 1 mg/ml RNase for 5 min at room temperature and then stained with propidium iodide at 100 μg/ml to determine DNA cell cycle using a FACS Verse (BD Bioscience, San Jose, CA). Doublets, cell debris, and fixation artifacts were gated out, and DNA cell cycle was determined using FACSuite Version 1.0.2.2238.

### Mouse experiments

The experimental protocol was approved by the Ethics Review Committee for Animal Experimentation of Kawasaki University Graduate School of Medicine and Dentistry (Ethics Committee reference number: 15–046). EBC2 lung SCC cells were plated in 15 cm dishes at a density of 4 × 10^6^ per dish and cultured overnight at 37°C. The following day cells were infected with Ad-null, Ad-sh*SOX2* or Ad-ATF/*SOX2* at a MOI of 250 for 24 hours. Cells were harvested and resuspended in culture medium. Human lung cancer xenografts were established in 6-wk-old female BALB/c nude mice (CLEA Japan, Inc., Tokyo, Japan) by subcutaneous (s.c.) inoculation of the adenoviral treated EBC2 cells (2 × 10^6^ cells/0.1 ml) into the dorsal flank. Fifteen mice were studied in each group. Animals were then observed closely and survival studies were performed. Tumors were measured 2 times a week, and tumor volume was calculated as a × b^2^ × 0.5, where a and b were large and small diameters, respectively.

### Statistical analysis

Statistically significant differences between means and medians of the study groups were evaluated using Student’s *t*-test (two-tailed, unpaired). Statistical significance was defined as ^*^p < 0.01.

## SUPPLEMENTARY MATERIALS FIGURES



## Abbreviations

SCC: squamous cell carcinoma
ATF: artificial transcription factor
bp: base pair
sh*SOX2*: *SOX2* short hairpin RNA
NSCLC: Non-small cell lung cancer
NGS: next generation sequencing
EGFR: epidermal growth factor receptor
ALK: anaplastic lymphoma kinase
HFF1: human foreskin fibroblast
NHLF: normal human lung fibroblast
HUVEC: human umbilical vein endothelial cells
ATFs: artificial transcription factors
DRPs: designed regulatory proteins
CPPs: cell-penetrating peptides
NLS: nuclear localization signal
UTR: untranslated region
MOI: multiplicity of infection
BLAST: Basic local alignment search tool
bp: base pair
EMT: epithelial-mesenchymal transition
RNAi: RNA interference
siRNA: small interfering RNAs
TALE: transcription activator-like effector
CRISPR: clustered regularly interspaced short palindromic repeat
CRISPR a/i: CRISPR activation and interference
GFP: green fluorescence protein
