# Supplementary material for: Targeted silencing of SOX2 by an artificial transcription factor showed antitumor effect in lung and esophageal squamous cell carcinoma
Source: Oncotarget. 2017 Oct 5;8(61):103063–76. doi: 10.18632/oncotarget.21523 (PMC5732711; doi:10.18632/oncotarget.21523)
Supplement: Supplementary file 1 [file oncotarget-08-103063-s001.pdf]

Targeted silencing of SOX2 by an artificial transcription factor showed antitumor effect in lung and esophageal squamous cell carcinoma

SUPPLEMENTARY MATERIALS

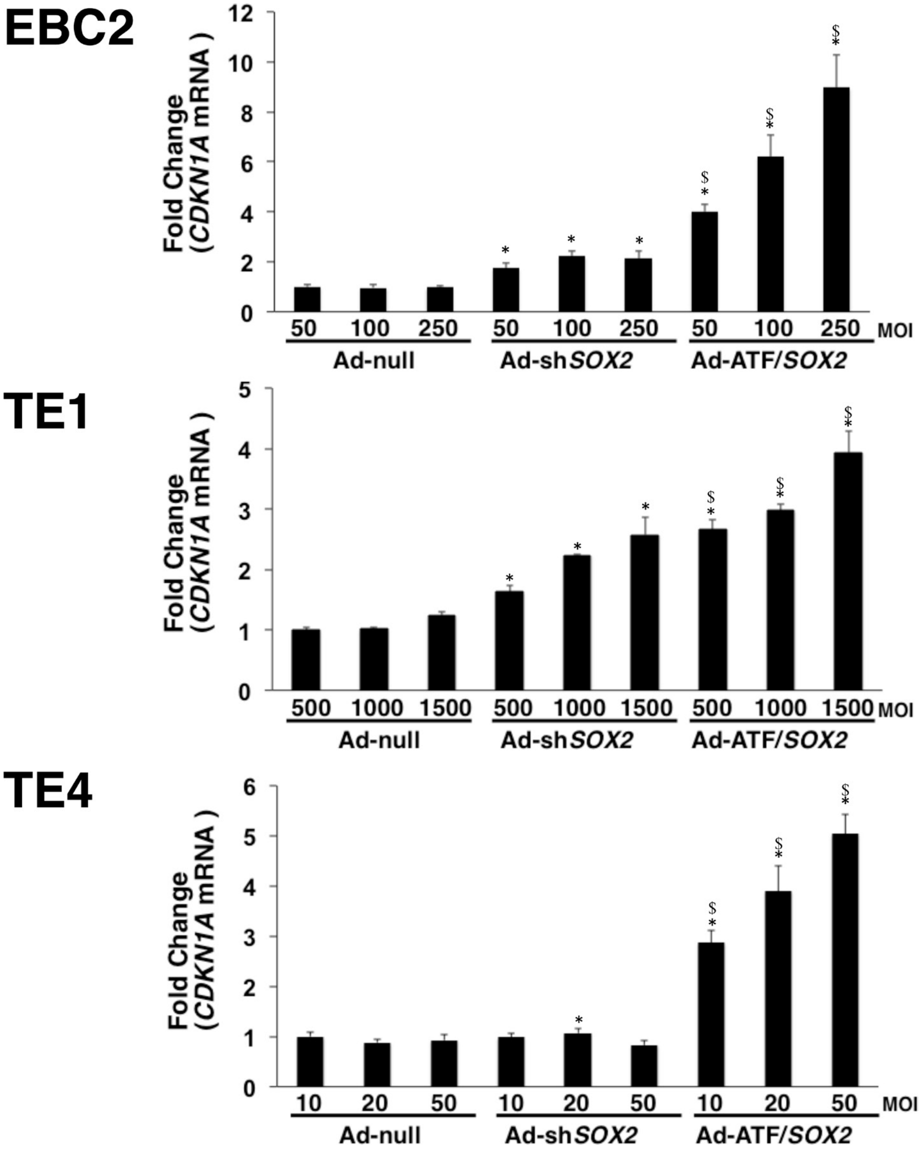

Supplementary Figure 1: Ad-ATF/*SOX2* up-regulated CDKN1A mRNA in lung and esophageal SCC cells more than Ad-sh*SOX2*. qPCR analysis showed that Ad-ATF/*SOX2* increased CDKN1A mRNA expression more significantly than Ad-sh*SOX2* 48 hours after adenoviral infections in all kinds of cells indicated. Fold changes relative to the CDKN1A mRNA induced by Ad-null at the minimal MOI in each kinds of cells were represented. Results represent the mean ± SD ($n = 3$). Statistical analysis was performed using Student's t test (two-tailed, unpaired). Statistical significance was defined as $^{*}p < 0.01$ vs Ad-null treated group at the same MOI; $^{\$}p < 0.01$ vs Ad-sh*SOX2* treated group at the same MOI.

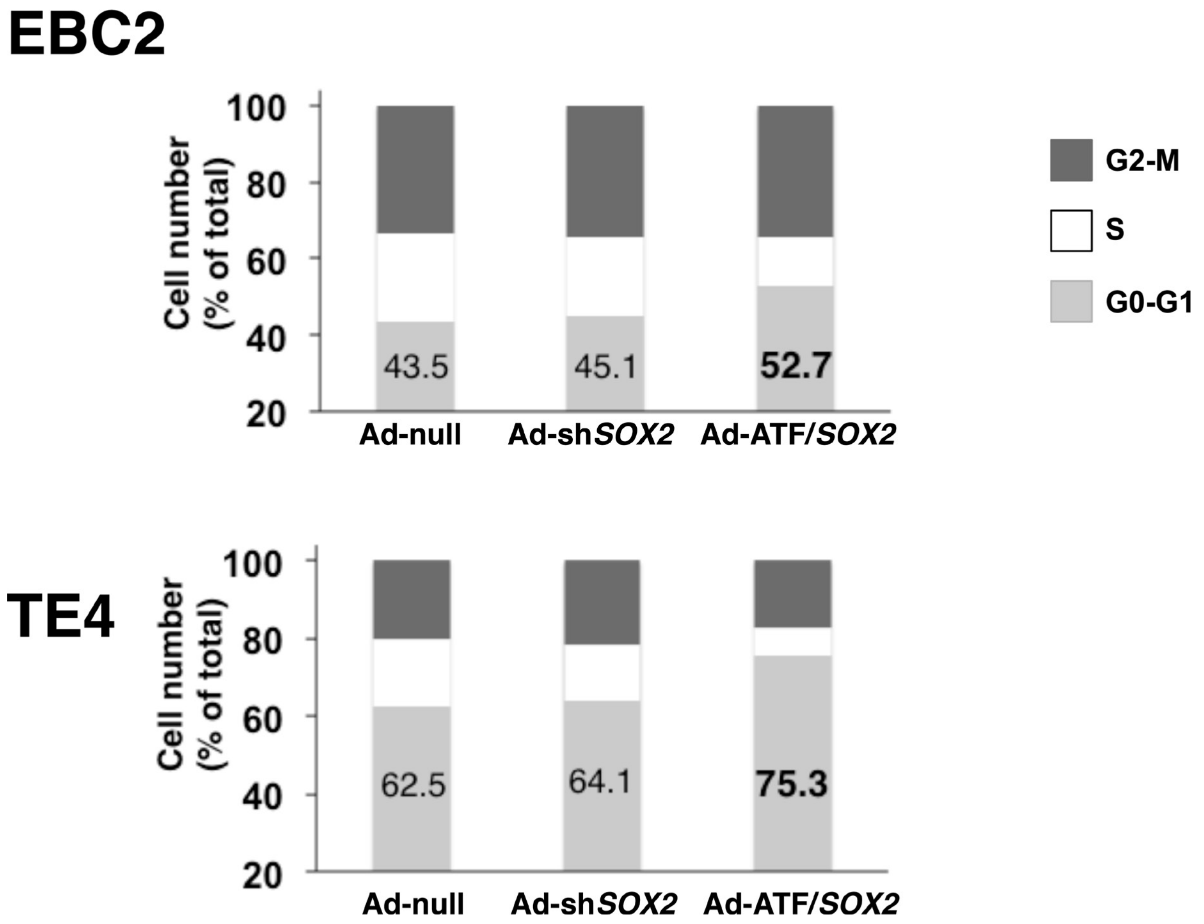

Supplementary Figure 2: Ad-ATF/*SOX2* increased G0-G1 population in EBC2 lung SCC cells and TE4 esophageal SCC cells. Flow cytometric cell cycle analysis of EBC2 lung SCC cells and TE4 esophageal SCC cells 36 hours after indicated adenoviral infections. The cell distribution at each phase is drawn in histograms.

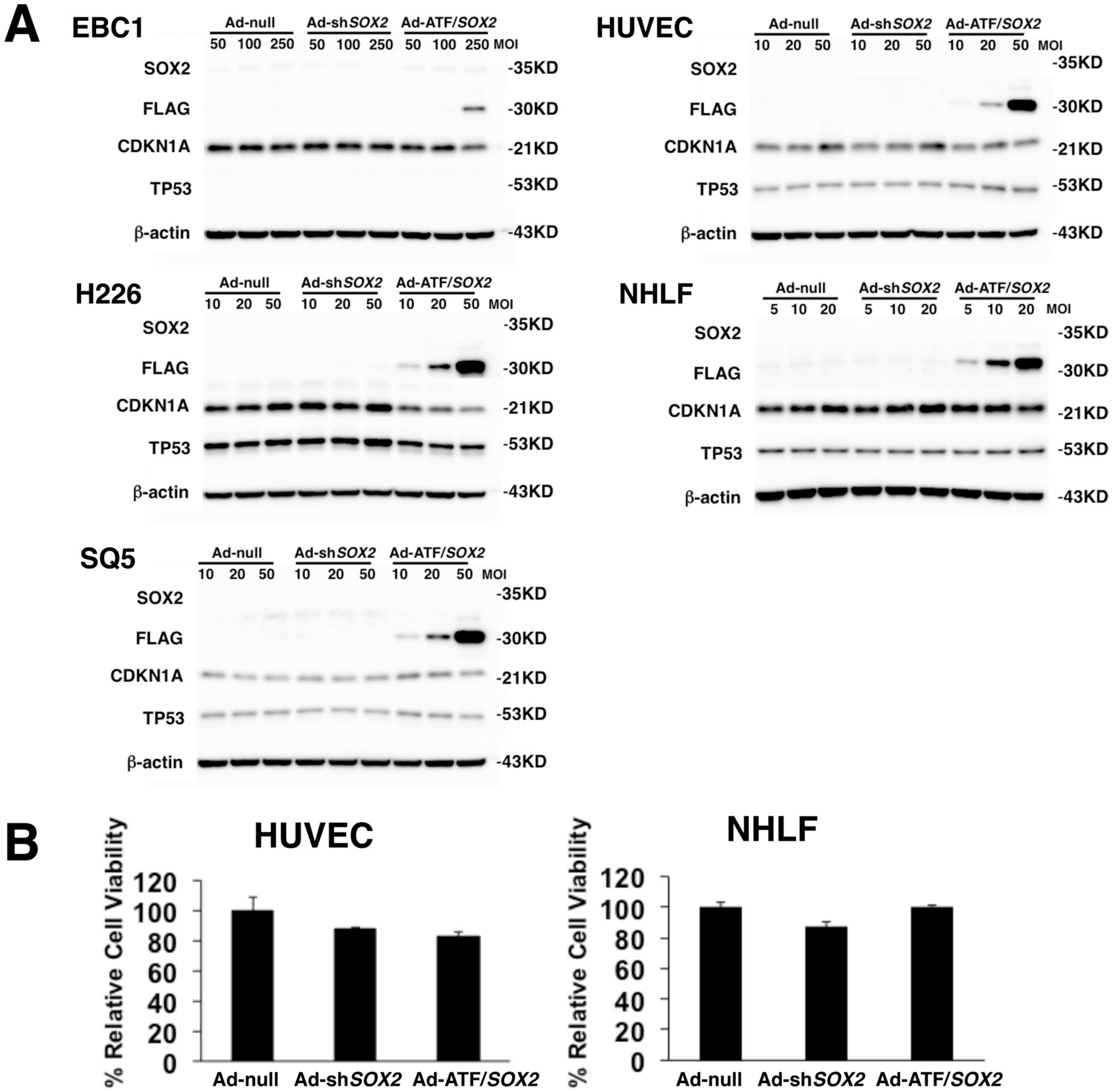

Supplementary Figure 3: Ad-ATF/*SOX2* induced little CDKN1A expression in normal cells HUVEC and NHLF. (**A**) Immunoblot analysis shows Ad-sh*SOX2* and Ad-ATF/*SOX2* increased little CDKN1A expression 48 hours after adenoviral infections in *SOX2* negative lung SCC cells and normal human cells. (**B**) Ad-ATF/*SOX2* did not significantly inhibit cell growth of HUVEC and NHLF cells that lack SOX2 expression compared to control ($p > 0.01$ vs Ad-null treated group). Cell viability was assessed 48 hours after adenoviral infection with a TC20 automated cell counter. Results represent the mean ± SD ($n = 3$).

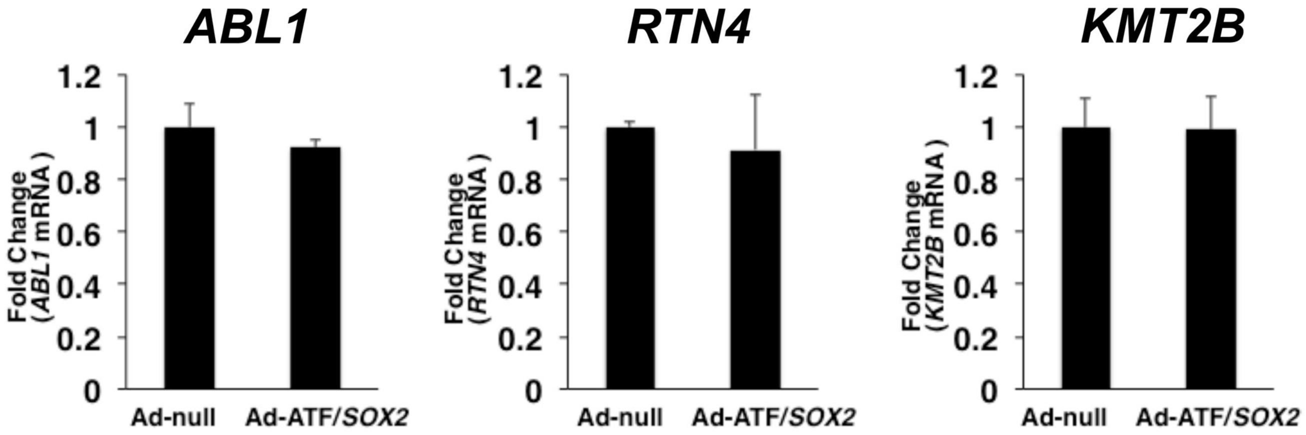

Supplementary Figure 4: Ad-ATF/*SOX2* did not change mRNA expression of ABL1, RTN4 and KMT2B that contained highly similar sequence to the 19 bp target sequence of ATF/SOX2 in EBC2 cells. Nucleotide Blast revealed that Homo sapiens ABL proto-oncogene 1, non-receptor tyrosine kinase transcript variant a mRNA (ABL1, NM_005157.5), Homo sapiens reticulon 4 transcript variant 1 mRNA (RTN4, NM_020532.4) and Homo sapiens lysine methyltransferase 2B mRNA (KMT2B, NM_014727.2) had high-identity matched sequence to the 19 bp target sequence of ATF/*SOX2* (more than 16 base-pair match with 100% identity and no sequence gaps). qPCR showed that their mRNA expression 48 hours after Ad-ATF/*SOX2* infection was not significantly changed compared to that after Ad-null infection.

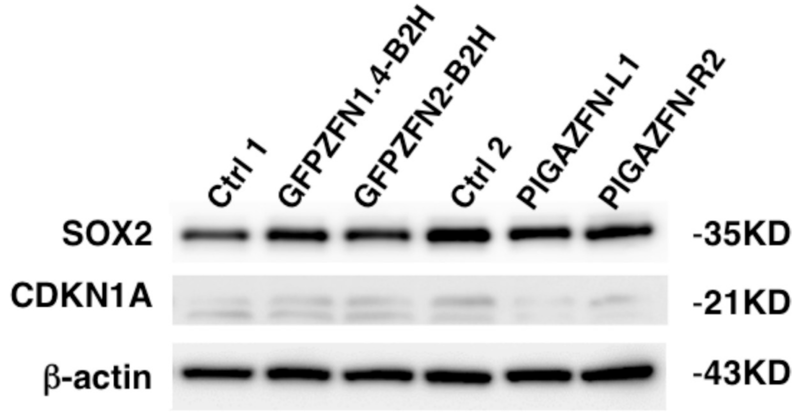

Supplementary Figure 5: The other kinds of zinc finger based transcription factors did not alter SOX2 or CDKN1A expression in EBC2 lung squamous cells. Immunoblot showed that SOX2 and CDKN1A expression was not changed 72 hours after transfection with plasmid vectors that express the other kinds of zinc finger based transcription factors (pGFPZFN1.4-B2H, pGFPZFN2-B2H, pPIGAZFN-L1 and pPIGAZFN-R2 in EBC2 cells. Ctrl 1: pcDNA3.1, Ctrl 2: pST1374. Protein was isolated after puromycin selection as described in Materials and Methods section.

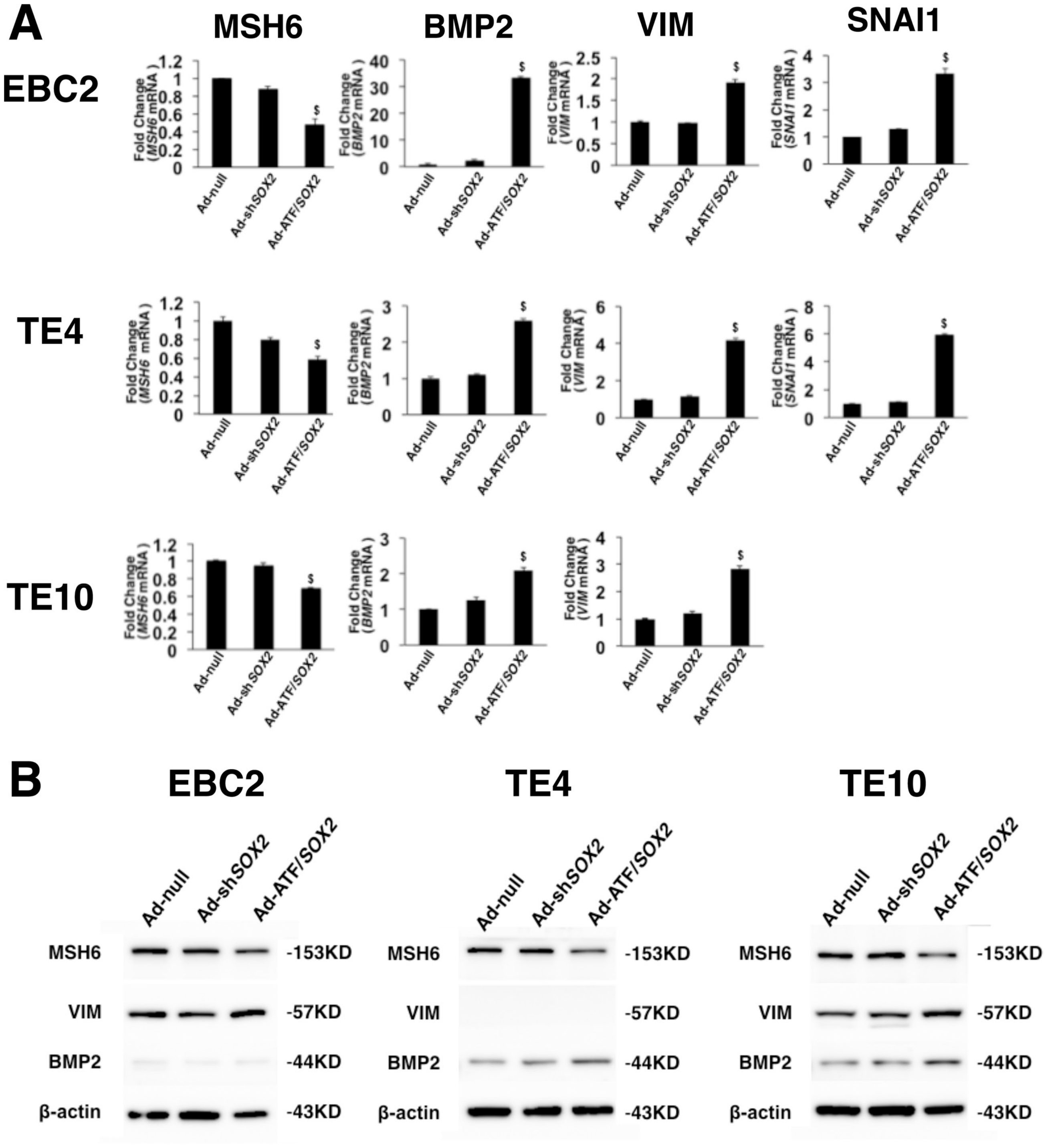

Supplementary Figure 6: ATF/SOX2 altered MSH6, BMP2 and VIM expression in lung and esophageal SCC cells. (**A**) qPCR demonstrated that mRNA expression of MSH6 mRNA was significantly down-regulated in EBC2 cells, TE4 cells and TE10 cells 48 hours after Ad-ATF/*SOX2* infection. On the other hand, BMP2 and VIM mRNA were up-regulated in all these kinds of cells 48 hours after infection. Another EMT related gene; SNAI1 mRNA was increased in EBC2 cells and TE4 cells 72 hours and 48 hours after Ad-ATF/*SOX2* infection respectively. SNAI1 mRNA was not increased in TE10 cells after Ad-ATF/*SOX2* infection (data not shown). Detection of GAPDH was used for normalization. Results represent the mean ± SD (n = 3). $^{\$}p < 0.01$ vs Ad-sh*SOX2* treated group. (**B**) Immunoblot analysis showed that MSH6 was decreased 72 hours after Ad-ATF/*SOX2* infection in all kinds of cells indicated.

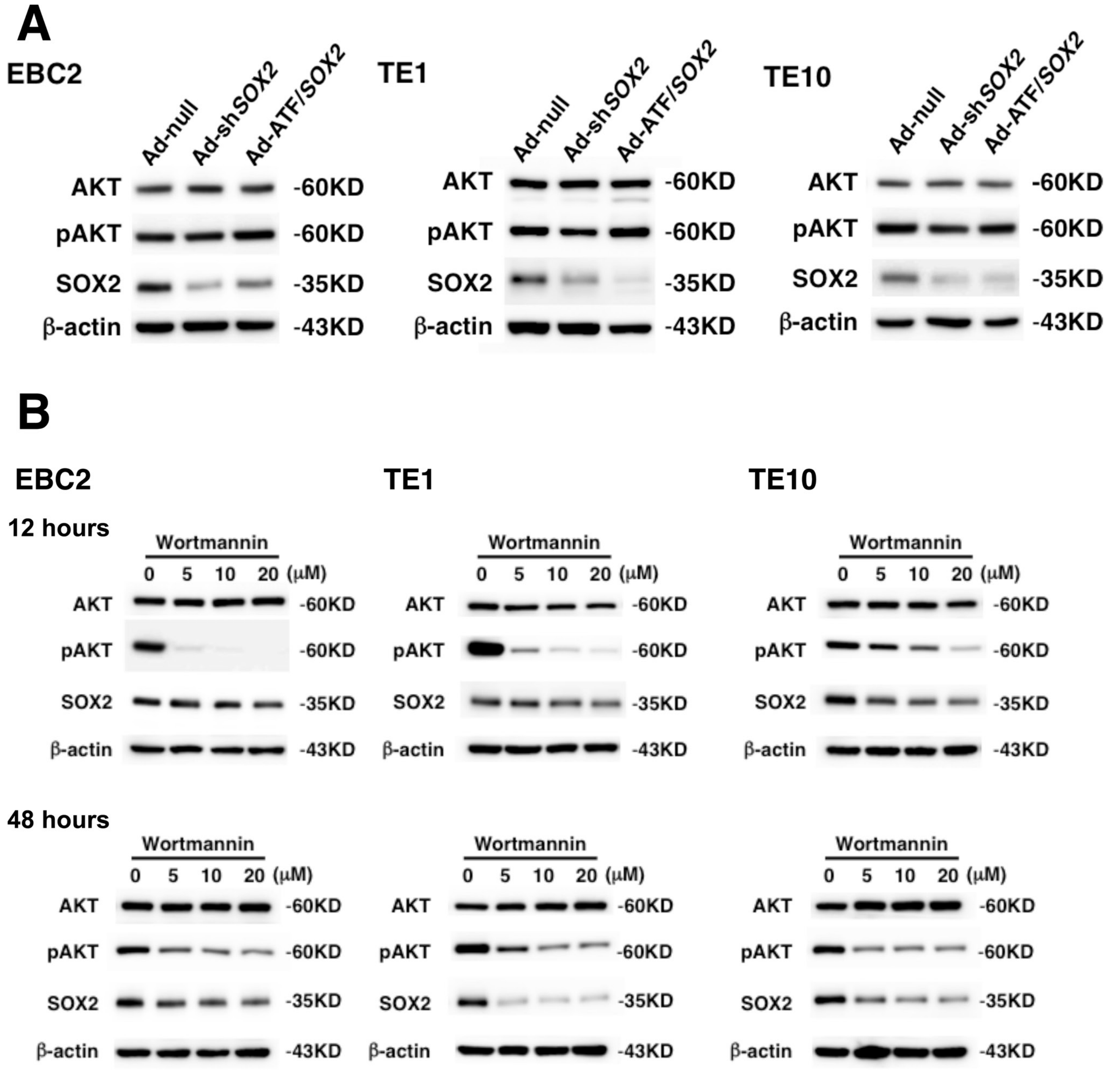

Supplementary Figure 7: PI3 Kinase inhibitor, wortmannin suppressed SOX2 expression in lung and esophageal SCC cells. (**A**) Immunoblot analysis demonstrated that Ad-shSOX2 and Ad-ATF/SOX2 did not alter phosphorylated AKT (pAKT) expression in EBC2 lung SCC cells and TE1 and TE10 esophageal SCC cells 48 hours after infection. (**B**) Wortmannin dose dependently suppressed pAKT and SOX2 expression in EBC2 cells, TE1 cells and TE10 cells. These results indicate that AKT might be an upstream regulator of SOX2 expression in lung and esophageal SCC cells.

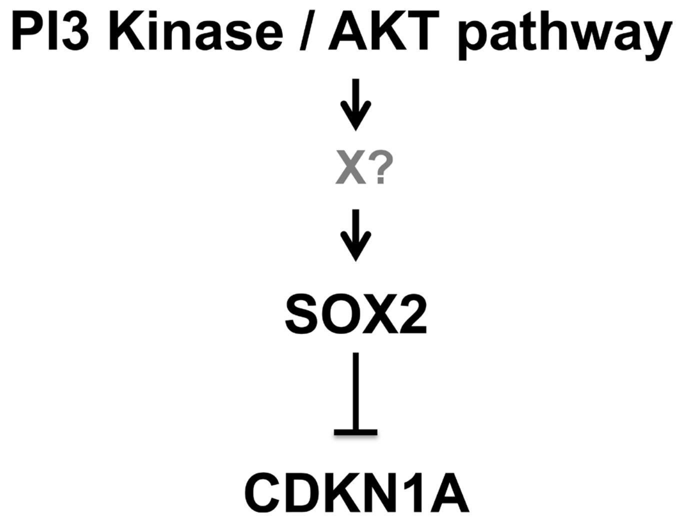

Supplementary Figure 8: Schematic illustration to show how SOX2 regulates the proliferation of lung and esophageal cancer cells.
